# Karyotype-specific cardiovascular and metabolic profiles in Turner syndrome: a retrospective echocardiographic study

**DOI:** 10.1186/s13023-026-04457-5

**Published:** 2026-06-20

**Authors:** Yinchun Huang, Song Luo, Yiying Qi, Shuang Qin, Chaomin Yue, Qinsheng Lu, Gendie E. Lash, Li Li

**Affiliations:** 1 Department of Obstetrics and Gynecology, Guangzhou Women and Children’s Medical Center, Guangzhou Medical University, Jin Sui Road 9, Guangzhou, Guangdong Province 510623 China; 2 Division of Uterine Vascular Biology, Guangzhou Institute of Pediatrics, Guangzhou Women and Children’s Medical Center, Guangzhou Medical University, Jin Sui Road 9, Guangzhou, Guangdong Province 510623 China

**Keywords:** Turner syndrome, Karyotype, Cardiovascular disease, Echocardiography, 45,X, Mosaicism, Dyslipidemia, Aortic size index

## Abstract

**Background:**

Turner syndrome (TS) is associated with cardiovascular abnormalities and metabolic risk, but karyotype-specific phenotypes remain incompletely defined, particularly in pediatric and adolescent cohorts assessed by transthoracic echocardiography (TTE).

**Methods:**

We conducted a retrospective cross-sectional study of 289 patients with TS evaluated between October 2016 and October 2023. Patients were grouped as 45,X monosomy (*n* = 149) or mosaic/structural karyotypes (*n* = 140). Baseline or preoperative TTE data were used. The primary endpoint was a clinically relevant structural cardiovascular disease (CVD) composite based on explicit baseline/preoperative diagnoses of major lesions; patent foramen ovale, isolated persistent left superior vena cava, and minor valve regurgitation were treated as descriptive findings. Aortic dilatation based on ASI > 20 mm/m² was analyzed separately as an aortic-size phenotype.

**Results:**

The structural CVD composite was more frequent in the 45,X group than in the mosaic/structural group (49/149 [32.9%] vs. 26/140 [18.6%], *p* = 0.006). In multivariable logistic regression, 45,X remained associated with the structural CVD composite after adjustment for age, BMI, systolic blood pressure, LDL-C, and HOMA-IR (OR 1.88, 95% CI 1.05–3.35; *p* = 0.033). Aortic dilatation by ASI > 20 mm/m² did not differ significantly between groups (28.9% vs. 32.9%, *p* = 0.462). Elevated total cholesterol was more frequent in the 45,X group, and LDL-C was independently associated with the structural CVD composite. LVDD was modestly lower in the 45,X group (33.6 ± 6.6 vs. 35.4 ± 6.4 mm; *p* = 0.017).

**Conclusions:**

In this single-center TS cohort, 45,X monosomy was associated with a higher burden of clinically relevant structural cardiovascular abnormalities and an adverse lipid profile, whereas ASI-defined aortic dilatation alone did not differ significantly by karyotype. These findings support karyotype-aware cardiovascular surveillance, while underscoring the need for cautious interpretation of small TTE differences and validation in multicenter longitudinal studies.

## Introduction

Turner syndrome (TS) is a rare chromosomal disorder caused by complete or partial loss of one sex chromosome and is associated with short stature, gonadal dysgenesis, metabolic abnormalities, and increased cardiovascular morbidity [1–4]. Cardiovascular involvement in TS includes bicuspid aortic valve (BAV), coarctation of the aorta (CoA), aortic arch abnormalities, aortic dilatation, and other congenital heart defects [5–8]. Cardiovascular disease remains one of the major determinants of morbidity and mortality in TS [5, 9–11].

Current guidelines emphasize lifelong cardiovascular surveillance in TS [2, 3, 5, 12]. TTE is practical and widely available for initial evaluation and follow-up, whereas cardiac magnetic resonance imaging (CMR) remains important for comprehensive assessment of the thoracic aorta and complex congenital anatomy [5, 13–16]. The 2024 international TS guideline recommends TS-specific Z-score assessment for children younger than 15 years and indexed aortic measurements, including ASI, for adolescents and adults; the 2022 ACC/AHA aortic disease guideline also recommends TTE and cardiac MRI at diagnosis and ASI use in patients aged 15 years or older [2, 13]. 

The clinical phenotype of TS varies substantially by karyotype [17–22]. Complete 45,X monosomy has been associated in some studies with a greater burden of cardiovascular anomalies than mosaic or structural karyotypes, but findings across cohorts are not fully consistent [17–22]. In addition, metabolic variables such as dyslipidemia and insulin resistance may modify cardiovascular risk [23–26]. The present study was designed to address the clinically relevant question of whether baseline cardiovascular and metabolic phenotypes differ between 45,X monosomy and mosaic/structural karyotypes in a single-center TS cohort.

## Methods

### Study design and participants

This was a retrospective cross-sectional study of patients with TS evaluated at Guangzhou Women and Children’s Medical Center between October 2016 and October 2023. The analysis included 289 patients with cytogenetically confirmed TS and available baseline clinical and TTE data. To maintain a focused baseline karyotype comparison, only baseline or preoperative TTE data were used in this manuscript; postoperative follow-up measurements were not analyzed.

### Karyotype classification

Peripheral blood lymphocyte karyotypes were determined by standard G-banding. Patients were categorized as 45,X monosomy (*n* = 149) or mosaic/structural karyotypes (*n* = 140). The mosaic/structural group included 45,X/46,XX, 45,X/46,XY, 45,X/47,XXX, isochromosome Xq, marker chromosome, and other structural sex chromosome abnormalities.

### Clinical, metabolic and endocrine variables

Baseline age, height, weight, BMI, BSA, heart rate, systolic and diastolic blood pressure, lipid variables, fasting glucose, fasting insulin, HOMA-IR, estradiol, and peak growth hormone were extracted from the medical record and laboratory databases. BSA was calculated using the Haycock formula [27]. Low estradiol was defined as < 73.4 pmol/L. Elevated total cholesterol was defined as > 5.2 mmol/L, elevated LDL-C as > 3.37 mmol/L, elevated triglycerides as > 1.70 mmol/L, and low HDL-C as < 1.30 mmol/L [28]. Elevated HOMA-IR was defined as > = 3.2 for patients younger than 18 years and > = 2.5 for patients aged 18 years or older [29–31].

### Echocardiographic assessment and cardiovascular endpoints

Baseline TTE parameters included AOD, aortic root Z-score when available, ASI, LAD, LVDD, LVDS, RVOT/RV diameter, MPAD, FS, EF, and valve peak velocities [15, 32, 33]. For patients who underwent cardiac surgery, the preoperative or earliest diagnostic TTE was used. ASI was calculated as AOD in millimeters divided by BSA in square meters; ASI > 20 mm/m² was analyzed as a separate aortic-size phenotype [2, 13, 34].

The primary endpoint was a clinically relevant structural CVD composite based on explicit baseline/preoperative diagnostic text and clinically adjudicated baseline data. The composite included BAV, CoA, aortic arch hypoplasia or arch obstruction, ASD, VSD, PDA, TOF, clinically relevant valve disease, and other clinically relevant cardiovascular abnormalities. PFO, isolated PLSVC, isolated arrhythmia, and minor or trace valve regurgitation were treated as descriptive findings and were not included in the primary composite. Aortic dilatation by ASI > 20 mm/m² was analyzed separately because ASI alone may be sensitive to body size in pediatric TS and should be interpreted in the context of age and imaging modality [2, 13].

### Statistical analysis

Continuous variables are reported as mean ± standard deviation and compared using Welch’s t test. Categorical variables are reported as n/N (%) and compared using chi-square or Fisher exact tests as appropriate. Multivariable logistic regression was used to assess the association between 45,X monosomy and the structural CVD composite after adjustment for age, BMI, SBP, LDL-C, and HOMA-IR. Linear regression was used for LVDD. Two-sided p values < 0.05 were considered statistically significant. Analyses were performed using Python/statsmodels and were cross-checked against the submitted source data.

## Results

The main results are summarized in Tables 1, 2, 3 and 4, including baseline clinical, metabolic, and endocrine characteristics (Table 1), cardiovascular endpoints and selected descriptive findings (Table 2), baseline transthoracic echocardiographic parameters (Table 3), and multivariable models (Table 4).

**Table 1:** Baseline clinical, metabolic, and endocrine characteristics by karyotype

Variable	45,X (*n* = 149)	Mosaic/structural (*n* = 140)	*p* value
Age (years)	8.6 ± 4.1	9.4 ± 4.7	0.160
Height (cm)	112.5 ± 18.9	116.1 ± 18.3	0.107
Weight (kg)	29.9 ± 11.0	30.5 ± 11.9	0.633
BMI (kg/m²)	23.3 ± 5.2	22.3 ± 5.5	0.118
BSA (m²)	1.0 ± 0.2	1.0 ± 0.3	0.469
Heart rate (bpm)	91.1 ± 13.4	92.0 ± 14.0	0.570
SBP (mmHg)	121.2 ± 14.2	119.2 ± 14.9	0.247
DBP (mmHg)	78.8 ± 10.9	77.2 ± 12.2	0.241
Total cholesterol (mmol/L)	4.6 ± 1.2	4.5 ± 1.0	0.307
LDL-C (mmol/L)	3.2 ± 1.5	2.9 ± 1.2	0.060
Triglycerides (mmol/L)	1.8 ± 1.5	1.5 ± 1.2	0.080
HDL-C (mmol/L)	1.7 ± 0.6	1.7 ± 0.6	0.401
Estradiol (pmol/L)	77.4 ± 56.7	80.4 ± 53.2	0.643
Peak GH (ng/mL)	1.5 ± 1.9	1.9 ± 4.3	0.418
HOMA-IR	2.4 ± 2.9	2.3 ± 2.1	0.681
Elevated total cholesterol	42/149 (28.2)	22/139 (15.8)	0.012
Elevated LDL-C	50/148 (33.8)	33/139 (23.7)	0.061
Elevated triglycerides	42/149 (28.2)	34/139 (24.5)	0.473
Low HDL-C	30/146 (20.5)	25/140 (17.9)	0.564
Low estradiol (< 73.4 pmol/L)	93/149 (62.4)	88/140 (62.9)	0.938
GH deficiency	144/147 (98.0)	133/138 (96.4)	0.490
Elevated HOMA-IR	29/149 (19.5)	34/140 (24.3)	0.321

Values are mean ± SD or n/N (%). BSA was calculated by the Haycock formula [27]. Low estradiol was defined as < 73.4 pmol/L. HOMA-IR, homeostasis model assessment of insulin resistance

**Table 2:** Cardiovascular endpoints and selected descriptive findings by karyotype

Endpoint	45,X (*n* = 149)	Mosaic/structural (*n* = 140)	*p* value
Primary clinically relevant structural CVD composite	49/149 (32.9)	26/140 (18.6)	0.006
Aortic/left-sided structural lesions	16/149 (10.7)	6/140 (4.3)	0.039
Aortic dilatation by ASI > 20 mm/m² (separate aortic-size phenotype)	43/149 (28.9)	46/140 (32.9)	0.462
Bicuspid aortic valve	4/149 (2.7)	4/140 (2.9)	1.000
Coarctation of the aorta	11/149 (7.4)	3/140 (2.1)	0.053
Aortic arch hypoplasia/arch obstruction	2/149 (1.3)	1/140 (0.7)	1.000
Atrial septal defect	14/149 (9.4)	5/140 (3.6)	0.046
Ventricular septal defect	17/149 (11.4)	5/140 (3.6)	0.012
Patent ductus arteriosus	9/149 (6.0)	9/140 (6.4)	0.891
Tetralogy of Fallot	1/149 (0.7)	1/140 (0.7)	1.000
Clinically relevant valve disease	14/149 (9.4)	10/140 (7.1)	0.488
Other clinically relevant cardiovascular findings	14/149 (9.4)	9/140 (6.4)	0.352
Patent foramen ovale (descriptive)	21/149 (14.1)	12/140 (8.6)	0.140
Persistent left superior vena cava (descriptive)	15/149 (10.1)	4/140 (2.9)	0.017

The primary structural CVD composite excluded PFO, isolated PLSVC, isolated arrhythmia, and minor/trace valve regurgitation. ASI-defined aortic dilatation was analyzed separately as an aortic-size phenotype

**Table 3:** Baseline transthoracic echocardiographic parameters by karyotype

TTE parameter	45,X (*n* = 149)	Mosaic/structural (*n* = 140)	*p* value
AOD (mm)	16.4 ± 4.0	17.0 ± 3.4	0.226
ZAOD	0.08 ± 2.47	0.34 ± 2.14	0.342
ASI (mm/m²)	18.0 ± 6.2	18.3 ± 6.4	0.669
LAD (mm)	19.5 ± 3.8	20.1 ± 4.2	0.173
RVOT/RV (mm)	14.5 ± 3.2	15.2 ± 3.2	0.072
LVDD (mm)	33.6 ± 6.6	35.4 ± 6.4	0.017
LVDS (mm)	21.6 ± 4.2	22.3 ± 4.2	0.115
MPAD (mm)	16.8 ± 3.7	17.0 ± 3.4	0.666
FS (%)	38.0 ± 28.4	36.7 ± 3.9	0.590
EF (%)	65.9 ± 6.9	67.4 ± 4.8	0.033
PV Vmax (m/s)	1.23 ± 1.95	0.95 ± 0.29	0.093
AV Vmax (m/s)	1.30 ± 1.99	1.14 ± 0.45	0.341
MV Vmax (m/s)	1.30 ± 2.25	1.03 ± 0.24	0.191
TV Vmax (m/s)	0.95 ± 2.16	0.74 ± 0.14	0.231

Values are mean ± SD. Baseline/preoperative TTE data were used. ZAOD values were taken from the source echocardiographic data when available

**Table 4:** Multivariable models for cardiovascular and echocardiographic outcomes

Outcome	Predictor	Adjustment model	Effect estimate (95% CI)	*p* value	*N*
Clinically relevant structural CVD composite	45,X vs. mosaic/structural	Model 1: age, BMI, SBP	2.01 (1.15 to 3.53)	0.015	288
Clinically relevant structural CVD composite	45,X vs. mosaic/structural	Model 2: age, BMI, SBP, LDL-C, HOMA-IR	1.88 (1.05 to 3.35)	0.033	286
Clinically relevant structural CVD composite	LDL-C, per 1 mmol/L	Model 2: age, BMI, SBP, LDL-C, HOMA-IR	1.37 (1.12 to 1.67)	0.002	286
Aortic dilatation by ASI > 20 mm/m²	45,X vs. mosaic/structural	Model 1: age, BMI, SBP	0.79 (0.45 to 1.39)	0.409	288
Aortic/left-sided structural lesions	45,X vs. mosaic/structural	Model 1: age, BMI, SBP	2.50 (0.94 to 6.64)	0.066	288
LVDD (mm)	45,X vs. mosaic/structural	Model: age, BSA	-1.78 (-3.29 to -0.28)	0.021	288

ORs are reported for logistic regression models; beta coefficients are reported for linear regression. BMI, body mass index; SBP, systolic blood pressure; LDL-C, low-density lipoprotein cholesterol

### Study population and karyotype distribution

Among 289 patients with TS, 149 were categorized as 45,X monosomy and 140 as mosaic/structural karyotypes. Baseline clinical, metabolic, and endocrine characteristics are shown in Table 1. The two groups were similar with respect to age, height, weight, BMI, BSA, heart rate, SBP, and DBP.

### Metabolic and endocrine profiles

The 45,X group showed a less favorable lipid profile. Elevated total cholesterol was more frequent in the 45,X group than in the mosaic/structural group (28.2% vs. 15.8%, *p* = 0.012). Elevated LDL-C showed a similar trend (33.8% vs. 23.7%, *p* = 0.061), although the between-group difference did not reach statistical significance in the current adjudicated dataset. Low estradiol, GH deficiency, and elevated HOMA-IR did not differ significantly between karyotype groups.

### Cardiovascular phenotypes

Cardiovascular phenotype distributions are shown in Table 2. The primary clinically relevant structural CVD composite was more frequent in the 45,X group than in the mosaic/structural group (32.9% vs. 18.6%, *p* = 0.006). Aortic/left-sided structural lesions were also more frequent in the 45,X group (10.7% vs. 4.3%, *p* = 0.039). CoA, ASD, VSD, and PLSVC were numerically or statistically more frequent in the 45,X group, whereas ASI-defined aortic dilatation did not differ significantly between groups (28.9% vs. 32.9%, *p* = 0.462).

### Baseline TTE parameters

Baseline TTE findings are summarized in Table 3. AOD, ZAOD, ASI, LAD, LVDS, MPAD, and valve peak velocities did not differ significantly between groups. LVDD was modestly lower in the 45,X group (33.6 ± 6.6 vs. 35.4 ± 6.4 mm, *p* = 0.017). EF was slightly lower in the 45,X group, although mean values in both groups were within a preserved range. These small differences should be interpreted cautiously and do not establish diastolic dysfunction.

### Adjusted associations

Multivariable models for cardiovascular and echocardiographic outcomes are summarized in Table 4. 45,X monosomy remained associated with the structural CVD composite after adjustment for age, BMI, SBP, LDL-C, and HOMA-IR (OR 1.88, 95% CI 1.05–3.35; *p* = 0.033). LDL-C was independently associated with the structural CVD composite (OR 1.37 per 1 mmol/L, 95% CI 1.12–1.67; *p* = 0.002). In contrast, 45,X was not associated with ASI-defined aortic dilatation after adjustment. In linear regression adjusted for age and BSA, 45,X was associated with a lower LVDD (beta − 1.78 mm, 95% CI -3.29 to -0.28; *p* = 0.021).

**Fig. 1:**
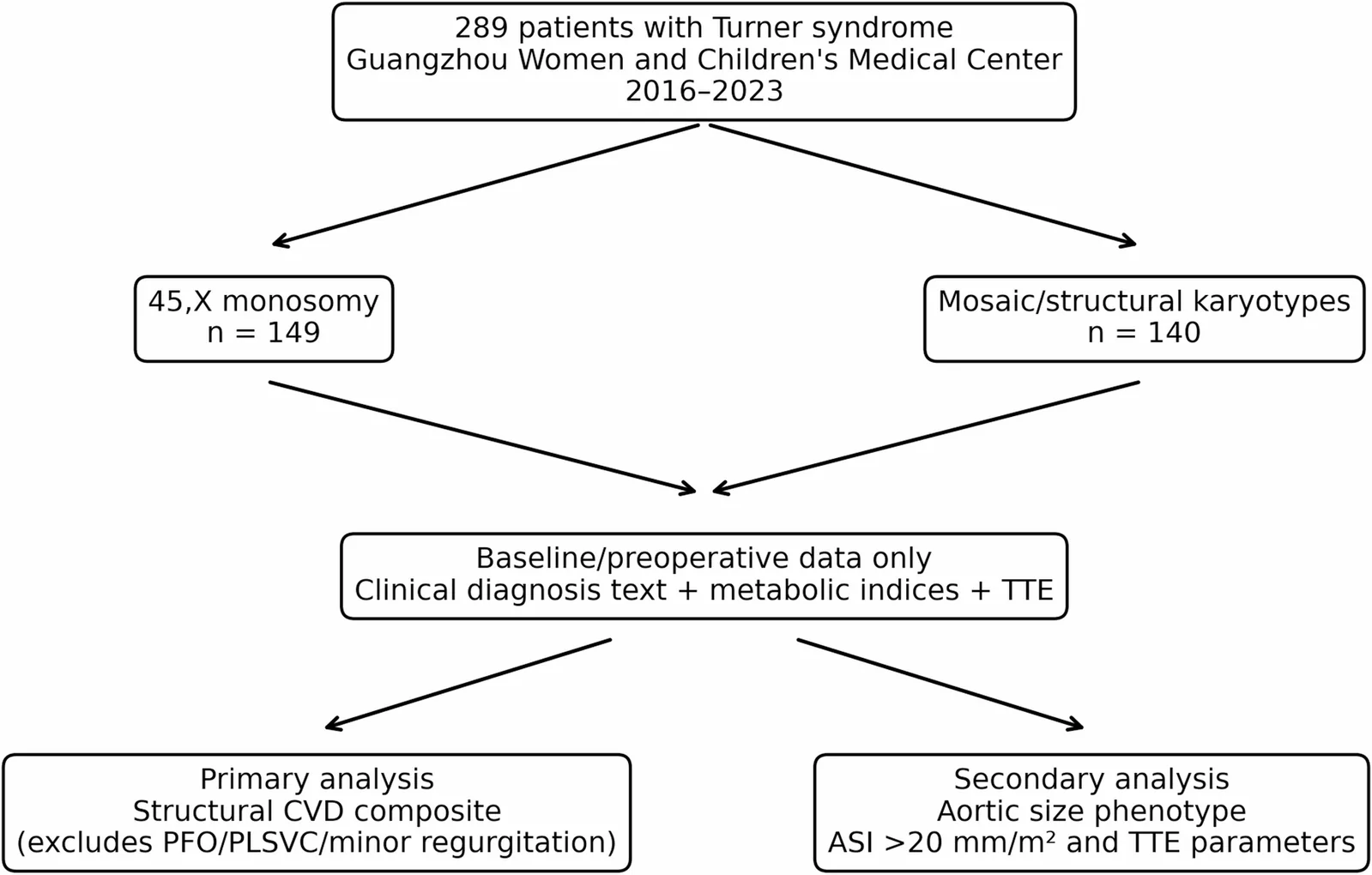
Study flowchart and analytic framework. The revised analysis included 289 patients with Turner syndrome and focused on within-TS karyotype comparisons. Baseline/preoperative TTE data were used; postoperative recovery data were not analyzed in this manuscript

**Fig. 2:**
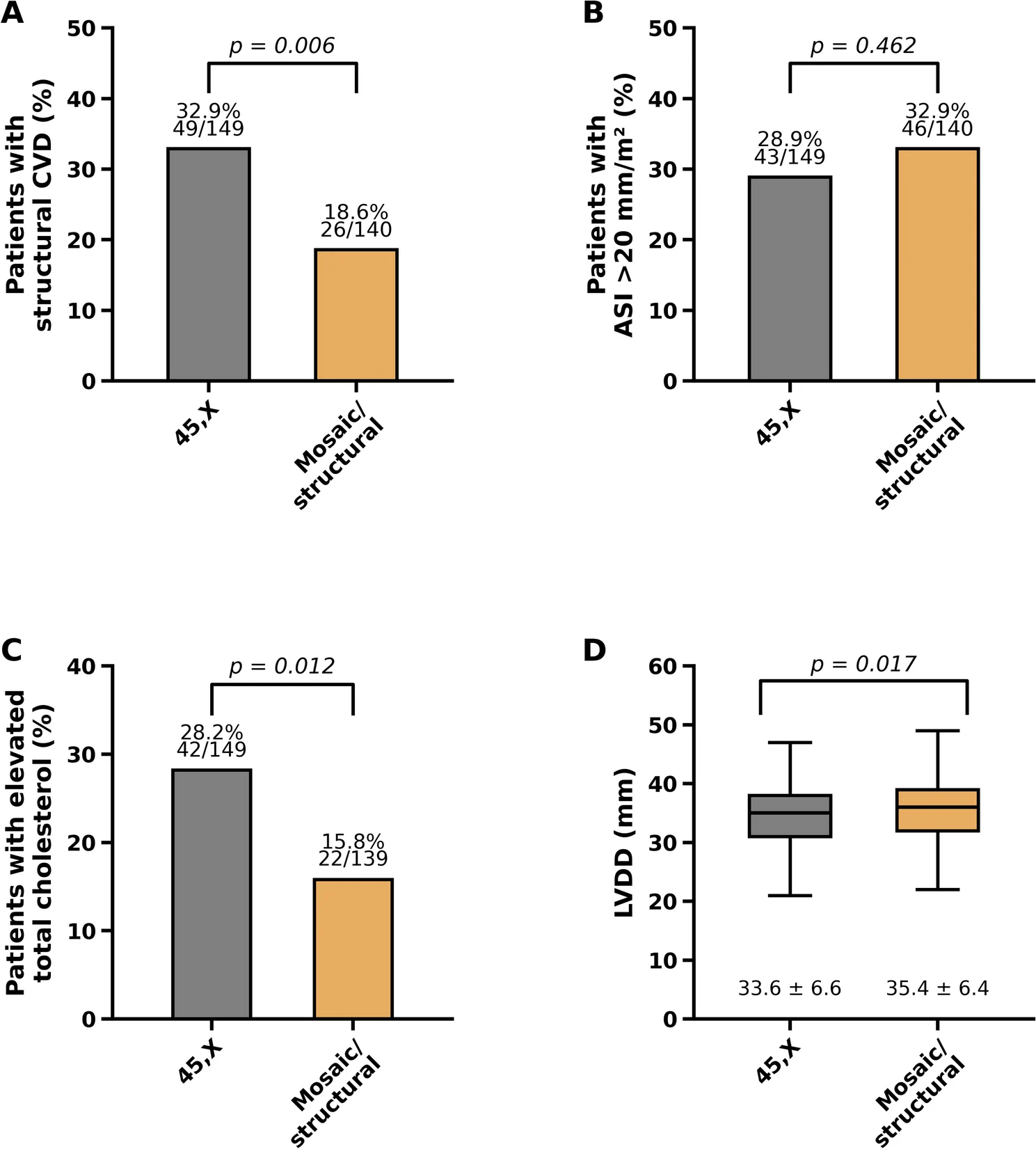
Key karyotype-specific cardiovascular and metabolic findings in patients with Turner syndrome. (**A**) The primary clinically relevant structural cardiovascular disease composite was more frequent in patients with 45,X monosomy than in those with mosaic/structural karyotypes. (**B**) Aortic dilatation defined by ASI > 20 mm/m² did not differ significantly between groups. (**C**) Elevated total cholesterol was more frequent in the 45,X group. (**D**) LVDD was modestly lower in the 45,X group; this small dimensional difference was not interpreted as evidence of diastolic dysfunction. Categorical variables were compared using chi-square or Fisher’s exact tests, and LVDD was compared using Welch’s t test. ASI, aortic size index; CVD, cardiovascular disease; LVDD, left ventricular end-diastolic dimension

The study flowchart and analytic framework are presented in Fig. 1, and the key karyotype-specific cardiovascular and metabolic findings are presented in Fig. 2.

## Discussion

This analysis provides a focused evaluation of karyotype-specific cardiovascular and metabolic profiles in TS. The principal findings are as follows. First, 45,X monosomy was associated with a higher burden of clinically relevant structural cardiovascular abnormalities than mosaic/structural karyotypes. Second, 45,X was associated with an adverse lipid profile, particularly elevated total cholesterol, and LDL-C was independently associated with the structural CVD composite. Third, ASI-defined aortic dilatation did not differ significantly by karyotype in this predominantly pediatric cohort. Fourth, the LVDD difference between groups was small and should not be interpreted as evidence of diastolic dysfunction.

The association between 45,X and clinically relevant structural CVD is consistent with reports suggesting a greater cardiovascular burden in complete monosomy than in mosaic or structural karyotypes [17–22]. However, the lack of a significant karyotype difference in ASI-defined aortic dilatation underscores the complexity of aortic assessment in pediatric TS [2, 13, 14, 16, 34]. Because ASI is influenced by BSA and many patients in this cohort were children, ASI-based findings should be interpreted alongside age, body size, TTE image quality, and where possible, CMR [2, 5, 13, 14, 16].

The lipid findings may be clinically relevant. Elevated total cholesterol was more common in the 45,X group, and LDL-C remained independently associated with the structural CVD composite. These observations support integrated cardiovascular and metabolic surveillance in TS, particularly in patients with complete monosomy [2, 5, 23–26].

The LVDD result requires caution. The difference was approximately 1.8 mm and may be within the range of measurement variability, especially in a pediatric retrospective TTE cohort [32, 33]. Therefore, we removed the previous statement that 45,X patients had impaired diastolic function. The revised conclusion is limited to a small LV dimension difference whose clinical significance remains uncertain.

This study has limitations. It was retrospective and single-center, and the cohort was predominantly pediatric, limiting generalizability to adult TS populations. CMR was not systematically available. Aortic assessment relied primarily on TTE and ASI, and should be interpreted in the context of age, body size, and imaging modality [2, 5, 13, 14, 16, 34]. Karyotype subtypes within the mosaic/structural group were heterogeneous, and sample size limited subtype-specific modeling. Finally, lesion-specific cardiovascular diagnoses were adjudicated from baseline or preoperative echocardiographic reports and clinical records, but external validation in multicenter cohorts remains necessary.

## Conclusions

In this single-center cohort of 289 patients with TS, 45,X monosomy was associated with a higher burden of clinically relevant structural cardiovascular abnormalities and an adverse lipid profile compared with mosaic/structural karyotypes. ASI-defined aortic dilatation did not differ significantly by karyotype. TTE showed a small LVDD difference, but this should not be overinterpreted as diastolic dysfunction. These findings support karyotype-aware cardiovascular and metabolic surveillance while emphasizing the need for prospective multicenter validation with standardized TTE and CMR-based aortic assessment.

## Data Availability

Data and materials are available from the corresponding author upon request.

## Abbreviations

AAH: Aortic arch hypoplasia
AOD: Aortic root diameter
ASI: Aortic size index
BAV: bicuspid aortic valve
BMI: Body mass index
BSA: Body surface area
CI: Confidence interval
CMR: Cardiac magnetic resonance imaging
CoA: Coarctation of the aorta
CVD: Cardiovascular disease
EF: Ejection fraction
FS: Fractional shortening
GH: Growth hormone
HDL-C: High-density lipoprotein cholesterol
HOMA-IR: Homeostasis model assessment of insulin resistance
LAD: Left atrial diameter
LDL-C: Low-density lipoprotein cholesterol
LVDD: Left ventricular end-diastolic dimension
LVDS: Left ventricular end-systolic dimension
MPAD: Main pulmonary artery diameter
PDA: Patent ductus arteriosus
PFO: Patent foramen ovale
PLSVC: Persistent left superior vena cava
SBP: Systolic blood pressure
TOF: Tetralogy of Fallot
TS: Turner syndrome
TTE: Transthoracic echocardiography
VSD: Ventricular septal defect
ZAOD: Z-score for aortic root diameter
